# Distribution of *Toxoplasma gondii* IgM and IgG antibody seropositivity among age groups and gestational periods in pregnant women

**DOI:** 10.12688/f1000research.15344.3

**Published:** 2019-06-18

**Authors:** Shahida Sadiqui, Syed Rafiq Hussain Shah, Babiker Saad Almugadam, Qismat Shakeela, Shehzad Ahmad

**Affiliations:** 1 Department of Microbiology, Hazara University, Mansehra, Pakistan; 2 Department of Microbiology, Faculty of Medical Laboratory Sciences, University of El Imam El Mahdi, Kosti city, White Nile state, Sudan; 3 Department of Microbiology, Abbottabad University of Science and Technology, Mansehra, Pakistan

**Keywords:** Toxoplasma gondii, Toxoplasmosis, Seroprevalence, IgG, IgM, Pregnant women

## Abstract

**Background**: Toxoplasmosis is a globally distributed parasitic disease. The present study aimed to estimate the prevalence and geographic distribution of toxoplasmosis as well as determine the percentage of toxoplasmosis-associated IgM and IgG seropositivity among different age groups. In addition, it aimed to estimate the proportion of toxoplasma IgM seropositivity among pregnancy trimesters.

**Methods**: A total of 500 pregnant women were included in this study. From each participant, a 5-ml venous blood sample was collected and centrifuged to obtain serum that was tested for Toxoplasma gondii IgM and IgG antibodies using immunochromatographic testing and ELISA.

**Results**: The overall seroprevalence of toxoplasmosis was 24.8%. Out of the total of 500 participants, only 8% had a serological marker of acute toxoplasmosis). There is a statistically significant difference in the seroprevalence of disease among the study areas. Amongst positive cases of every trimester, 54.34% of first trimester positive cases had a serologic marker for acute toxoplasmosis.

**Conclusions**: In this study, there is a high prevalence of toxoplasmosis. Therefore, it is necessary to test every pregnant woman for toxoplasmosis and distinguish the type of infection, as well as the conduction of public health education programs to generate the awareness.

## Introduction

Toxoplasmosis is a widely distributed zoonotic illness causes by *Toxoplasma gondii*, an obligate intracellular parasite ^1, 2^. Globally, the distribution of this disease is extremely variable even inside the countries ^3, 4^. In all host species, including humans, Toxoplasmosis is generally acquiring either vertically from mother to fetus (congenital infection), or through ingestion of oocysts in contaminated food or water ^5^. Rarely, *T. gondii* can transmit through organ transplantation and the transfusion of infected blood ^6, 7^. Following ingestion, the intestinal epithelium is the primary portal of entrance for *T. gondii*; next, it spreads to other tissues, where it can cause more severe pathogenesis ^8, 9^. If toxoplasmosis is acquired during pregnancy, severe infection may develop, especially in immunocompromised individuals, such as those with defects in T-cell-mediated immunity ^10^. In patients with AIDS, toxoplasmosis may lead to life-threatening disease ^11^. For example, cerebral focal lesions are caused by cerebral toxoplasmosis (CT) in HIV-infected patients ^12^.

The signs and symptoms of this illness are markedly divergent and range from asymptomatic to serious infection ^13^. This variation depends on several factors includes inoculums size, virulence of the strain of toxoplasma, the individual’s genetic background and the status of the immune system of the infected individual ^14^. In addition, since the organism has an affinity for muscular and neural tissues as well as the other visceral organs, many hosts harboring latent tissue cysts following toxoplasmosis ^15^.

Fetuses may acquire toxopasmosis through the placenta during pregnancy ^16^. Early infection of the fetus may cause severe damage, or either pre- or post-natal death ^17^. The clinical manifestations of congenital toxoplasmosis generally depends on the gestational stage, and can include seizures, mental retardation, severe neurological defects, chorioretinitis, epilepsy and blindness ^10, 16, 18^.

Approximately 90% of pregnant women infected with *T. gondii* are asymptomatic, and recover spontaneously ^19, 20^. Only a small percentage of pregnant women show the clinical symptoms of disease ^19, 21^. In pregnant women, the clinical signs are no more severe than in non-pregnant women, and typically an influenza-like illness is seen after an incubation period of 5 to 18 days ^19, 22, 23^. Early diagnosis and treatment of mothers during pregnancy prevents fetal infection and minimizes the probability of complications ^24, 25^.

Laboratory diagnosis of toxoplasmosis is usually performed by serological detection of *T. gondii*-specific IgG and IgM antibodies ^26^. Worldwide, the screening of *T. gondii* infection in pregnant women is preferably performed during the first trimester and subsequently every month or trimester in seronegative women, as applied in many countries ^27^.

Our study was undertaken to determine the prevalence and geographic distribution of toxoplasmosis as well as to estimate the seropositivity of toxoplasma antibodies among different age groups. It also attempted to identify the percentage of toxoplasma IgM seropositivity (indicative of acute infection) among different pregnancy trimesters.

## Methods

This a descriptive cross-sectional hospital-based study carried out in the District Head quarter Hospital (Mansehra, Hazara, Pakistan) and Ayub Medical Complex Hospital (Abbottabad, Khyber Pakhtunkhwa, Pakistan) over a period of 4 months (April to July 2015).

### Study population and sample size

Our study included pregnant women of different trimesters, ages and ethnic groups who visited our study areas hospitals; the only eligibility criteria were pregnancy and visiting the hospitals in our study area. Patients were recruited by the researchers face-to-face. During this study duration, a total of 500 pregnant women (convenience sample) fulfilled the inclusion criteria. Out of the total of participants, 204 were recruited from Abbottabad and 296 from Mansehra district.

### Laboratory analysis

A total of 5 ml venous blood was collected from each participant using a sterile syringe and transferred to a blood container without anticoagulant, allowed to clot at room temperature for 15 minutes, then centrifuged at 3000 rpm for 10 minutes to obtain serum, which was transferred into a 1.5ml microcentrifuge tube and stored at −80°C for further analysis. In this study, every sample was screened and confirmed for toxoplasmosis through the serological tests.

### Screening

All sera samples were screened for *T. gondii* IgG and IgM antibodies using Rapid Diagnostic immunochromatographic test (Tox IgG/IgM Rapid Test Dip strip, CTK BIOTECH, San Diego, USA) according to manufacturer instructions. In order to avoid false-positive results due to the incomplete specificity of the screening test, every positive sample was further subject to confirmation step by ELISA. Each positive individual also answered a questionnaire concerning their age, trimester and whether they had been in recent contact with animals ( Supplementary File 1).

### Confirmation

Following the screening, all the positive samples (n=150) were further confirmed to toxoplasmosis using IgM and IgG ELISA kit (Monobind, San Diego, USA) according to the manufacturer protocol. The positive ELISA test for *T. gondii* IgG titers indicates the chronic infection, whereas with high IgM titers indicate the recent or acute infection. All ELISA tests were performed in triplicate.

### Ethical statement

Our study was approved by the Ethics Review Committee of Hazara University. Further approval was provided by the administration of Ayub Medical Complex Hospital. From every participant, written informed consent was obtained for conduction of the study. In addition, all the performed steps in this study were completely in accordance with the Helsinki Declaration and the rules defined by the World Medical Association, including samples collection and processing.

### Statistical analysis

The obtained results were analyzed by Graph Pad Prism 5 (Graph Pad Software, La Jolla, CA, USA). A χ ^2^ test was involved to check the statistical differences in seropositivity and negativity of anti-toxoplasma antibodies among the participants of different study areas and gestational periods, at 95% level of significance. Moreover, ANOVA has tested the statistical difference of these antibodies among the participants of every age group. The difference was considered statistically significant when P <0.05.

## Results

### Seroprevalence of toxoplasmosis

**Figure 1.**
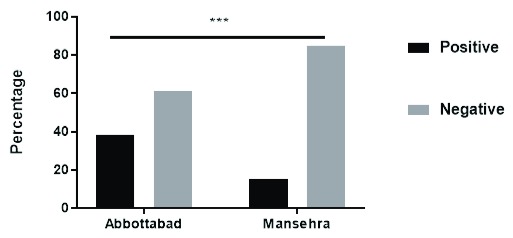
Seroprevalence of toxoplasmosis in different districts. Out of the total of participants in every district, 38.7% (79/204) had the serologic marker of toxoplasmosis in Abbottabad district and 15% (45/296) in Mansehra. ***P = 0.0002.

**Figure 2.**
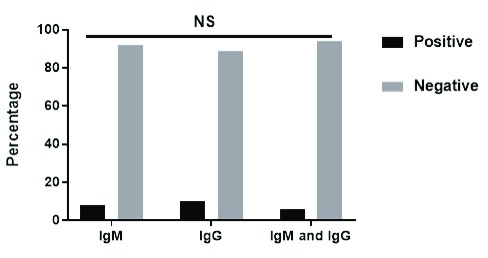
The overall prevalence of Toxoplasma IgM (acute infection) and IgG (chronic infection). Out of 500 pregnant women, 8% (40/500) were positive to IgM, 10.8% (54/500) to IgG, and 6% (30/500) to both antibodies. P = 0.567.

Out of 500 women, using ELISA the overall seroprevalence of toxoplasmosis was 24.8% (124/500). Statistically significant differences were observed between the seroprevalence of disease in Abbottabad and Mansehra district ( Figure 1). In addition, the prevalence of toxoplasma antibodies among pregnant women revealed out of the total of 500 participants, only 8% had a serological marker of acute toxoplasmosis ( Figure 2).

### Toxoplasma antibodies seropositivity among age groups and gestational periods in overall positive cases

**Figure 3.**
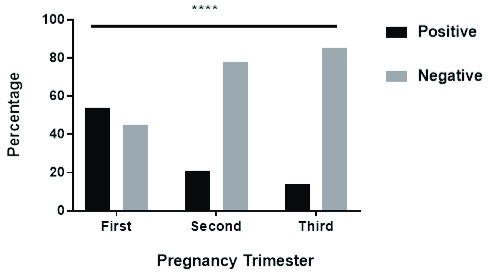
Percentage of IgM seropositivity among the total of positive cases in each pregnancy trimesters. Among the total of positive cases in every trimester, the seropositivity of IgM revealed statistically significant difference. Out of 46, 51, and 27 toxoplamosis infected cases in a first, second and third trimesters, respectively, 54.34% (25/46) were seropositive to IgM (acute infection) in first trimester, 21.56% (11/51) seropositive to IgM in second trimester, and 14.81% (4/27) seropositive to IgM in third trimester. ****P = 0.0001.

Among the positive cases (n=124), the seropositivity of toxoplasma antibodies was shown to be statistically significant different among different age groups ( Table 1). There was also a statistically significant difference in the seropositivity of toxoplasma IgM (indicating acute infection) between different gestational trimesters, the highest level of IgM seropositivity was observed in first trimester (54.34%) ( Figure 3).

**Table 1.** Percentage of *Toxoplasma gondii* antibodies seropositivity among the total of positive cases in every age group.

Age, years	Positive cases	IgG	IgM	IgG and IgM
17–24	46	43.5% (20/46)	32.6% (15/46)	23.9% (11/46)
25–32	54	40.7% (22/54)	35.2% (19/54)	24.1% (13/54)
33–40	24	50% (12/24)	25% (6/24)	25% (6/24)
**P value**		0.003		

The raw data associated with this study. Excel file includes the results of screening (ICT) and confirmatory tests (ELISA), plus the pregnancy trimesters of toxoplasmosis positive cases Click here for additional data file. Copyright: © 2019 Sadiqui S et al. 2019 Data associated with the article are available under the terms of the Creative Commons Zero "No rights reserved" data waiver (CC0 1.0 Public domain dedication).

## Discussion

Toxoplasmosis in pregnancy can predispose the fetus to serious complications ^28^. The fetus can be severely damaged when the infection is acquired during pregnancy ^29^. Therefore, testing the serum of pregnant women for toxoplasma IgG and IgM is important to avoid intrauterine infection and complications. The current study was conducted on 500 blood samples collected from pregnant women in Mansehra and Abbottabad district of Pakistan, and examined for *T. gondii* IgM (acute infection) and IgG (chronic infection) antibodies. Out of the total of 500 pregnant women, 24.8% (124 women) had a serologic marker of toxoplasmosis. Among the 124 positive cases, 54 were seropositive for toxoplasma IgG antibody, 40 cases for Toxo-IgM and 30 cases for both IgM and IgG antibody. In addition, out of 500 participants, 8% had a serologic marker of acute toxoplasmosis. In 2007, Obeed reported the prevalence of IgG (chronic infection) and IgM (acute infection) antibodies were 36% and 26.6%, respectively, which are greater than those seen in our study results ^30^. In addition, the seroprevalence of toxoplasmosis in Saudi Arabia was reported as 21.8% ^31^. In pregnant women from South Korea, a low prevalence was observed (0.79%) ^32^, with rates of 20% reported in Finland ^33^ and 24% in Prague ^34^. These findings indicate the prevalence of toxoplasmosis is markedly difference in different countries.

Moreover, our study revealed that the geographic distribution of toxoplasmosis is significantly different among the study areas. Out of the 296 participants analyzed from Mansehra and 204 from Abbottabad, the overall prevalence of toxoplasmosis was 15% and 38.7%, respectively. The higher prevalence in Abbottabad when compared with Mansehra may because Abbottabad is an area where agricultural practices are common, and domestic animals like cats and goats were generally kept in or near the homes. Thus, contact with these animals may be the main risk factor of the disease. In addition, low educational and socioeconomic level may have contributed.

In our study, a high percentage of IgM seropositivity was reported in the 1st trimester, which indicated a high prevalence of acute toxoplasmosis or recent infection in this trimester compared with the others. Furthermore, as reported in this study, there is a mild difference in the seropositivity of toxoplasma antibodies among age groups, which requires further study to assess whether, is there any significant association exists between toxoplasmosis and age.

Usually *T. gondii* does not cause clinical illness in the majority of animal species ^35^. Human often acquires this infection from animals by ingestion of improperly cooked or raw animal meat, or via consumption of contaminated food and water with animal’s waste ^14^. However, there is a need for detailed knowledge about the risk factors of toxoplasmosis. Previously, it was reported that some risk factors are associated with toxoplasmosis, such as owning cats ^36^. Additionally, the previous study revealed that that contact with domestic animals may associate with this disease ^37, 38^. Therefore, the next study studies should evaluate the role of cats contact in disease development.

In this study, a high prevalence of toxoplasmosis was revealed. Moreover, in the first and second trimester of pregnancy, the prevalence of acute toxoplasmosis seems to be higher compare with a third. Thus it is necessary to test every pregnant woman for toxoplasmosis and distinguish the type of infection. In addition, urgent treatment and medicine are essential to decrease the risk of intra-uterine infection and congenital toxoplasmosis. Additionally, there is a need to conduct public health education to create greater awareness about the disease, its transmission, symptoms, and prevention. In addition, screening of *T. gondii* infection and maternal care should be considered as the main stratagem to reduce the risks of congenital toxoplasmosis.

## Data availability

The data referenced by this article are under copyright with the following copyright statement: Copyright: © 2019 Sadiqui S et al.

Data associated with the article are available under the terms of the Creative Commons Zero "No rights reserved" data waiver (CC0 1.0 Public domain dedication). 

**Dataset 1. The raw data associated with this study.** Excel file includes the results of screening (ICT) and confirmatory tests (ELISA), plus the pregnancy trimesters of toxoplasmosis positive cases. .

DOI: https://doi.org/10.5256/f1000research.15344.d249392 ^39^.

## Supplementary material

**Supplementary File 1. Study questionnaire**
