# Supplementary material for: Distribution of Toxoplasma gondii IgM and IgG antibody seropositivity among age groups and gestational periods in pregnant women
Source: F1000Res. 2019 Jun 18;7:1823. Originally published 2018 Nov 20. [Version 3] doi: 10.12688/f1000research.15344.3 (PMC6584968; doi:10.12688/f1000research.15344.3)
Supplement: Supplementary file 2 [file f1000research-7-21380-s0001.tgz › 08c7a6aa-77f3-41e5-b3d6-26a06fbe20ec_Supplementary_File_1._Study_questionaire_V2.docx]

**Hazara University**

**Questionnaire**

# Distribution of *Toxoplasma gondii* IgM and IgG antibody seropositivity among age groups and gestational periods in pregnant women

**Personal and Medical information**

- Patient name ___________________
- Sample No ___________________
- Date ___________________
- City _______________________
- Age____________
- Pregnancy trimester ___________
- Previous History of animals contact Yes 􀁔 No 􀁔

Name of animal____________________________________________________

**Laboratory investigations**

- Screening (ICT): Seropositive 􀁔 Seronegative 􀁔

Seropositive for IgM 􀁔 IgG IgM and IgG 􀁔

- Confirmation (ELISA): Positive 􀁔 Negative 􀁔
