# Supplementary material for: Distribution of Toxoplasma gondii IgM and IgG antibody seropositivity among age groups and gestational periods in pregnant women
Source: F1000Res. 2019 Jun 18;7:1823. Originally published 2018 Nov 20. [Version 3] doi: 10.12688/f1000research.15344.3 (PMC6584968; doi:10.12688/f1000research.15344.3)
Supplement: Peer review history

# Peer review: Reviewer response for version 3

**DOI:** 10.5256/f1000research.21380.r50029

**Published:** 2019-07-01

**Authors:** Wafa Babiker

**Affiliations:** 1 Department of Microbiology, Faculty of Medical Laboratory Sciences, University of Khartoum, Khartoum, Sudan

It's an acceptable improvement.

I confirm that I have read this submission and believe that I have an appropriate level of expertise to confirm that it is of an acceptable scientific standard.

# Peer review: Reviewer response for version 3

**DOI:** 10.5256/f1000research.21380.r50028

**Published:** 2019-06-19

**Authors:** Asghar Fazaeli

**Affiliations:** 1 Department of Medical Parasitology & Mycology, School of Medicine, Zanjan University of Medical Sciences, Zanjan, Iran

I read the new version of the Sadiqui *et al.* article. Almost all my comments were addressed and corrections have been made by the authors. So, I can confirm the latest version of the article.

I confirm that I have read this submission and believe that I have an appropriate level of expertise to confirm that it is of an acceptable scientific standard.

# Peer review: Reviewer response for version 2

**DOI:** 10.5256/f1000research.20454.r46160

**Published:** 2019-04-08

**Authors:** Asghar Fazaeli

**Affiliations:** 1 Department of Medical Parasitology & Mycology, School of Medicine, Zanjan University of Medical Sciences, Zanjan, Iran

The authors' response about association of animal contact and toxoplasmosis is not convincing. The following descriptions may help to this discussion: 1) Cat is the only animal as the definitive host for Toxoplasma gondii which can shed Toxoplasma oocysts becoming infective after a few days in environment; so this is the only animal species with possible association of its contact with toxoplasmosis. 2) Other animals like caw, buffalo, goats, and dogs may harbor the latent parasite (tissue cysts) inside their body tissues and cannot transmit to human or other animal by outer contact. 3) Even if this idea, possible association between these animal spp and Toxoplasma infection, could be proposed, the method of the present study is not suitable for this evaluation, as the study population contained only seropositive women, whereas no seronegative control groups with animal contacts were included in the study. So, the authors' aim to measure the association of these animal contact with toxoplasmosis is not basically scientific and the relevant data and discussion are not acceptable.

I confirm that I have read this submission and believe that I have an appropriate level of expertise to confirm that it is of an acceptable scientific standard, however I have significant reservations, as outlined above.

# Peer review: Reviewer response for version 2

**DOI:** 10.5256/f1000research.20454.r46159

**Published:** 2019-04-03

**Authors:** Wafa Babiker

**Affiliations:** 1 Department of Microbiology, Faculty of Medical Laboratory Sciences, University of Khartoum, Khartoum, Sudan

I think, it is an acceptable improvement.

I confirm that I have read this submission and believe that I have an appropriate level of expertise to confirm that it is of an acceptable scientific standard.

# Peer review: Reviewer response for version 1

**DOI:** 10.5256/f1000research.16719.r43385

**Published:** 2019-02-12

**Authors:** Asghar Fazaeli

**Affiliations:** 1 Department of Medical Parasitology & Mycology, School of Medicine, Zanjan University of Medical Sciences, Zanjan, Iran

The manuscript is written in a good structure and presents useful data including the rate of anti- *Toxoplasma* antibodies (IgG and IgM) in sera of pregnant women referred to hospitals of two districts in Pakistan. The manuscript however requires some corrections and modifications as follows:

1. The authors should consider that “toxoplasmosis” is not necessarily “illness or disease” as such they have mentioned in many parts of the manuscript. Toxoplasmosis is an infection which is mostly latent and does not lead to disease or illness in most of immunocompetent individuals. Also, some other issues in the Introduction are scientifically susceptible, e.g. toxoplasma acquisition in fetuses during delivery (mentioned in the 3 ^rd^ paragraph of the Introduction); to my knowledge, this issue is not documented in the literature. 
2. The basis of data arrangement, analysis and discussion about percentage of *Toxoplasma gondii *antibodies in different age groups (Table 1) is not valid. For this issue, all 500 study cases must be primarily divided into different age groups, then the percentage of seropositivity (IgG, IgM, IgG+IgM) in all initial samples of each group should be calculated and consequently compared and analyzed.
3. There is no scientific reason to indicate possible relation between *Toxoplasma* infection and contact with animals like caw, buffalo, goats, and dogs, except for cats which are a definitive host of *Toxoplasma* and shed oocysts. The only way of passing *Toxoplasma* infection from these animals to humans, is ingestion of undercooked meats or rarely through contact of open wounds with meats contaminated with bradyziotes, but not other types of contact with these animals or their wastes. So, this variable (animal contact) is not wise to be included in the manuscript and is recommended to be removed from all parts of the manuscript.
4. Miss-citation is the case for some *Toxoplasma* facts in the manuscript. For example, cerebral toxoplasmosis is referred to Kristiah (2009), which is not the right citation; while it was originally reported and discussed by other researchers, i.e. Luft and Remington (1988 ^1^) and Luft and Remington (1992 ^2^).
5. English writing correction is required in some parts of the manuscript text.

I confirm that I have read this submission and believe that I have an appropriate level of expertise to confirm that it is of an acceptable scientific standard, however I have significant reservations, as outlined above.

## References

[1] : AIDS commentary. Toxoplasmic encephalitis. *J Infect Dis*.1988;157(1) :1-6 312175810.1093/infdis/157.1.1

[2] : Toxoplasmic encephalitis in AIDS. *Clin Infect Dis*.1992;15(2) :211-22 152075710.1093/clinids/15.2.211

# Response

**Published:** 2019-03-16

**Authors:** Syed Rafiq Hussain Shah

**Affiliations:** HAZARA UNIVERSITY, Pakistan

Dear Dr. Asghar Fazaeli,

Most of your suggestions have been addressed in Version 2, while we didn't address points 2 and 3 due to the following reasons:

1. Because we aimed to estimate the percentage of toxoplasma antibodies seropositivity among positive cases. Therefore, we calculate them by dividing the frequency of Ab seropositivity/Number of positive cases.
2. Because toxoplasmosis is a zoonotic disease, thus it can transmit to humans from animals as we mentioned in Version 1. In addition, in past research, scholars have found that there is an association of cat contact with disease development, while the association of other kinds of animals with disease is poorly understood. Therefore, we aimed to evaluate the role of different kinds of animals in disease development. 

Thank you.

# Peer review: Reviewer response for version 1

**DOI:** 10.5256/f1000research.16719.r40932

**Published:** 2018-12-10

**Authors:** Wafa Babiker

**Affiliations:** 1 Department of Microbiology, Faculty of Medical Laboratory Sciences, University of Khartoum, Khartoum, Sudan

This article has studied toxoplasmosis, which is considered as one of the most common causes of abortion, stillbirth, intrauterine death, and congenital abnormalities. In addition, it’s a globally distributed disease.  

 The paper is well designed and clearly written; the results were presented accurately and the conclusion is supported by study findings. In my opinion, it’s perfect and scientifically acceptable. However for further improvement of the article, the authors should revise the English grammar.

I confirm that I have read this submission and believe that I have an appropriate level of expertise to confirm that it is of an acceptable scientific standard.

# Response

**Published:** 2019-03-19

**Authors:** Syed Rafiq Hussain Shah

**Affiliations:** HAZARA UNIVERSITY, Pakistan

Dear Dr. Wafa Babiker,

     

We have addressed your suggestion in Version 2. Thank you.
